# Tunnel Magnetoresistance Sensors with Magnetostrictive Electrodes: Strain Sensors

**DOI:** 10.3390/s16111902

**Published:** 2016-11-11

**Authors:** Ali Tavassolizadeh, Karsten Rott, Tobias Meier, Eckhard Quandt, Hendrik Hölscher, Günter Reiss, Dirk Meyners

**Affiliations:** 1 Institute for Materials Science, Kiel University, Kaiserstrasse 2, 24143 Kiel, Germany; eq@tf.uni-kiel.de; 2 Department of Physics, Bielefeld University, Universitätsstrasse 25, 33615 Bielefeld, Germany; rott@physik.uni-bielefeld.de (K.R.); reiss@physik.uni-bielefeld.de (G.R.); 3 Institute of Microstructure Technology, Karlsruhe Institute of Technology (KIT), Hermann-von-Helmholtz Platz 1, 76344 Eggenstein-Leopoldshafen, Germany; meier_tobias@email.gwu.edu (T.M.); hendrik.hoelscher@kit.edu (H.H.)

**Keywords:** tunnel magnetoresistance, inverse magnetostriction, strain sensors

## Abstract

Magnetostrictive tunnel magnetoresistance (TMR) sensors pose a bright perspective in micro- and nano-scale strain sensing technology. The behavior of TMR sensors under mechanical stress as well as their sensitivity to the applied stress depends on the magnetization configuration of magnetic tunnel junctions (MTJ)s with respect to the stress axis. Here, we propose a configuration resulting in an inverse effect on the tunnel resistance by tensile and compressive stresses. Numerical simulations, based on a modified Stoner–Wohlfarth (SW) model, are performed in order to understand the magnetization reversal of the sense layer and to find out the optimum bias magnetic field required for high strain sensitivity. At a bias field of −3.2 kA/m under a $0.2\times 10^{-3}$ strain, gauge factors of 2294 and −311 are calculated under tensile and compressive stresses, respectively. Modeling results are investigated experimentally on a round junction with a diameter of 30±0.2μm using a four-point bending apparatus. The measured field and strain loops exhibit nearly the same trends as the calculated ones. Also, the gauge factors are in the same range. The junction exhibits gauge factors of 2150±30 and −260 for tensile and compressive stresses, respectively, under a −3.2 kA/m bias magnetic field. The agreement of the experimental and modeling results approves the proposed configuration for high sensitivity and ability to detect both tensile and compressive stresses by a single TMR sensor.

## 1. Introduction

Research on strain sensing in nano- and microscale has evolved with a perspective on delivering miniaturized, integrated, and high-speed sensing devices. They are essential for applications that demand high strain sensitivity in small strain scales including force sensors, pressure sensors, and microcantilever technology [1,2]. Profiting from high sensitivity [3], high band-width [4], and miniaturization possibilities, magnetostrictive magnetoresistance (MR) sensors are a promising alternative to piezoresistive and piezoelectric strain sensors. In particular, magnetostrictive TMR sensors with CoFeB/MgO/CoFeB structures [3,5,6] offer more scalability compared to magnetostrictive giant magnetoresistance sensors [7,8,9,10] and higher gauge factors compared to AlO*_x_*-based TMR sensors with amorphous CoFeB [11], crystalline Co_50_Fe_50_ [12] and amorphous (Fe_90_Co_10_)_78_Si_12_B_10_ [13] electrodes. The CoFeB/MgO/CoFeB TMR sensors have been successfully incorporated into membranes for pressure sensing [5,14] and microcantilevers for atomic force microscopy (AFM) [6,15].

A magnetostrictive TMR sensor consists of an MTJ with a magnetostrictive sense layer. The magnetization of the sense layer is free to rotate under mechanical stress. The stress-induced magnetization change has been investigated experimentally for magnetostrictive FeCoBSi microdots [16] and magnetostrictive TMR pressure sensors [14] by magnetic force microscopy and optical Kerr effect measurements, respectively. In contrast to the sense layer, the ferromagnetic reference layer is magnetically stabilized. As a result, the angle *α* between the magnetization of the two layers alters upon the applied stress, leading to changes of the tunnel resistance, described by its angular dependence in MTJs [17]:

(1) $$R\left(\alpha \right)=\frac{R_{⊥}}{1+\frac{R_{ap}-R_{p}}{R_{ap}+R_{p}}\cos\left(\alpha \right)},$$

where $R_{p}$, $R_{⊥}$, and $R_{ap}$ stand for resistance values as $\alpha =0^{\circ }$, $90^{\circ }$, and $180^{\circ }$, respectively. This equation allows the correlation of the resistance change, caused by the applied stress, to the angle *α*. In addition to a highly magnetostrictive sense layer and a high MR effect amplitude, a high response from MR sensors to mechanical stress requires a proper configuration of the applied stress and induced anisotropies of the sense and reference layers. There have been only few studies based on total energy minimization to find out the optimum configuration [18] and the response of MR sensors [12,19].

**Figure 1:**
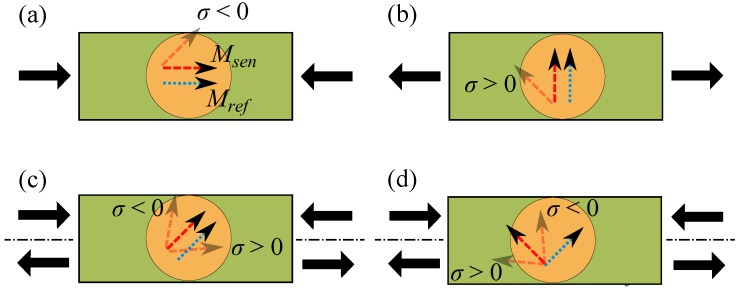
Different configurations of the easy axes of the sense and reference layers with respect to the stress axis leading to the detection of (**a**) compressive stress σ<0; (**b**) tensile stress σ>0; (**c**) compressive and tensile stresses and (**d**) compressive and tensile stresses with knowledge of the stress type. $M_{sen}$ (dashed red arrow) and $M_{ref}$ (dotted blue arrow) stand for the magnetization of the sense and reference layers, respectively. The orange circle shows a TMR sensor from the top placed on a cantilever (green rectangle). The black arrows indicate compressive and tensile stresses.

Up to now, MR-based strain sensors have been investigated in four different configurations [3,13,20] depicted in Figure 1. While the induced magnetic anisotropies or easy axes of the sense and reference layers are parallel (Figure 1a) or perpendicular (Figure 1b) to the stress axis, MR sensors only respond to compressive or tensile stress [13]. Aligning the two magnetic easy axes at 45${}^{\circ }$ toward the stress axis (Figure 1c), MR sensors become sensitive to both tensile and compressive stresses [20]. Additionally, with the last configuration (Figure 1d) the type of stress, whether tensile or compressive, can be also distinguished. The inverse impact on the angle *α* by tensile and compressive stresses results in an increase and a decrease of the tunnel resistance, respectively. This extends the applications further to detection of both tensile and compressive uniaxial stresses by a single sensor [3]. Such configuration is achievable, for instance, by internal couplings or an external bias magnetic field.

Following our recent study [3] of magnetostrictive TMR sensors with the configuration in Figure 1d, we extend the work here with a numerical modeling, based on a modified SW model of resistance versus field (R(H)) loops as TMR sensors are exposed to different stress or strain quantities. This leads to better understanding of the magnetization reversal of the sense layer and allows one to figure out the optimum bias field required for high strain sensitivity. Then, at the optimum bias field, resistance versus strain (R(ε)) loops are simulated to investigate the inverse influence on the tunnel resistance by tensile and compressive stresses. Results obtained by the modeling are experimentally studied on CoFeB/MgO/CoFeB-based TMR sensors by measuring field and strain loops. Finally, we briefly demonstrate the performance of these sensors in stress oscillation mode and their novel applications for dynamic AFM measurements.

## 2. Materials and Methods

### 2.1. Fabrication

**Figure 2:**
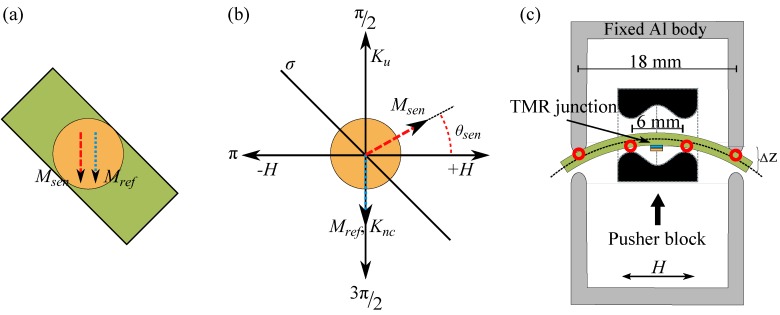
(**a**) The $45^{\circ }$ alignment of the induced anisotropy $K_{u}$ of the sense and reference layers with respect to the length of the cantilever or the stress axis. $M_{sen}$ and $M_{ref}$ of unstrained junctions stay parallel at the zero field due to the Néel coupling ($K_{nc}$) between the two layers; (**b**) Configuration of physical quantities with respect to $K_{u}$ and the stress axis. $\theta_{sen}$ denotes the orientation of $M_{sen}$; (**c**) Schematic of the four-point bending apparatus. The displacement of the pusher block ΔZ moves the ceramic pieces (black bodies) and imposes tensile or compressive stress into the TMR junction. The red circles depict the equidistant contact points.

A TMR stack was deposited by magnetron sputtering at a base pressure of $2\times 10^{-7}$ mbar on a 4 ″ Si (525±25μm)/SiO${}_{2}$ (1.5 μm) wafer purchased from Si-Mat Silicon Materials (Kaufering, Germany). The stack has the following multilayer structure (from bottom to top): Ta (5)/Ru (30)/Ta (10)/Ru (5)/MnIr (12)/CoFe (3)/Ru (0.9)/ CoFeB (3)/MgO (1.8)/CoFeB (3)/Ta (5)/Ru (5). The numbers in parentheses indicate the layer thicknesses in nm. At first, the stack was annealed at about 360 ${}^{\circ }$C for 1 h in a vacuum of $1\times 10^{-6}$ mbar under a magnetic field of 159.2 kA/m. This causes crystallization of the CoFeB layers and setting of the exchange bias across the MnIr/CoFe interface. As a result, the top Co${}_{40}$Fe${}_{40}$B${}_{20}$ layer with a high magnetostrictive coefficient [21] serves as the sense (free) layer whereas the bottom Co${}_{40}$Fe${}_{40}$B${}_{20}$ layer serves as the reference layer is magnetically stabilized by the pinned CoFe layer via interlayer exchange coupling. Then, the MTJ stack was structured into round junctions with nominal diameters of 11.3 μm to 41.8 μm in a sequence of standard lithography processes [22]. The junctions exhibit TMR effect amplitudes of ∼200% and resistance-area products of ∼550 kΩ·μm${}^{2}$. At the end, the wafer was diced into cantilevers with a size of 3 mm × 25 mm so that the uniaxial anisotropy $K_{u}$ induced during the field annealing process leads to an energetically favored alignment of the magnetization of the sense layer $M_{sen}$ and the reference layer $M_{ref}$ at $\frac{\pi }{4}$ toward the length of the cantilevers or the stress axis (Figure 2a).

### 2.2. Modeling

Assuming single-domain behavior, numerical simulations based on a modified SW model were performed to attain an understanding of $M_{sen}$ behavior within the R(H) and R(ε) loops. The shape and the size of the junctions were not taken into account. Considering magnetocrystalline anisotropy energy, Zeeman energy, Néel coupling energy, and magnetoelastic energy, the total energy density of the sense layer, $E(\theta_{sen})$, is determined by

(2) $$\begin{array}{cc} E(\theta_{sen}) & =K_{u}sin^{2}\left(\frac{\pi }{2}-\theta_{sen}\right)-\;\mu_{0}H_{}M_{s}cos\left(\theta_{sen}\right)-\;\mu_{0}H_{bias}M_{s}cos\left(\pi -\theta_{sen}\right)\\ & -K_{nc}cos\left(\frac{3\pi }{2}-\theta_{sen}\right)+K_{\sigma }sin^{2}\left(\theta_{\sigma }-\theta_{sen}\right). \end{array}$$

Here, $K_{u}$ given in Table 1 is calculated from the relation $K_{u}=\frac{\mu_{0}H_{k}M_{s}}{2}$ where $H_{k}=4$ kA/m and $M_{s}=1030$ kA/m are the magnetic anisotropy field and the saturation magnetization of the sense layer, respectively. $H_{k}$ was determined from fitting the hard-axis hysteresis curves [22]. $\theta_{sen}$ denotes the orientation of $M_{sen}$, and *H* is the sweeping magnetic field applied within the field loops. $H_{bias}$ stands for the bias magnetic field present during strain loops. $K_{nc}=\mu_{0}H_{sh}M_{s}$ is the Néel coupling anisotropy with $H_{sh}$ being the loop shift extracted from a standard TMR measurement [3]. $K_{\sigma }$ corresponds to the uniaxial stress-anisotropy given by $K_{\sigma }=\frac{3}{2}\lambda_{s}Y\varepsilon$ with $\lambda_{s}$ being the isotropic saturation magnetostriction, *Y* being the Young’s Modulus, and *ε* being the applied strain. Since the sense layer (CoFeB) is a positive magnetostrictive material, $K_{\sigma }$ is along $\theta_{\sigma }=\frac{3\pi }{4}$ for tensile stress whereas, in case of compressive stress, $K_{\sigma }$ resides at $\theta_{\sigma }=\frac{\pi }{4}$.

The energy minimization was carried out for every *H* or *ε* resolving the equilibrium states of $M_{sen}$ ($\theta_{sen}$) as *H* is swept or *ε* is ramped. Then, the equilibrium values of the angle *α* are calculated as $\alpha =\theta_{sen}-\theta_{ref}$ with $\theta_{ref}$ equal to $\frac{3\pi }{2}$ (Figure 2b). Using Equation (1), R(α) was determined for every equilibrium state, which resulted in the R(H) and R(ε) loops for *H* and *ε* being the variables, respectively. The resistance $R_{p}=0.78$ kΩ and $R_{ap}=2.23$ kΩ for the parallel and antiparallel magnetization configurations were considered for the calculation. They are experimental values of the junction resistance investigated in the experimental section. With these experimental values $R_{⊥}=1.16$ kΩ was deduced from Equation (1).

### 2.3. Experimental

In order to study the influence of mechanically applied uniaxial stress on the magnetostrictive TMR sensors on a cantilever, a four-point bending apparatus depicted in Figure 2c was used based on a so-called pusher block [9]. The cantilever is placed between four mechanical contact points. Homogenous straining is imposed to the cantilever by inner ceramic pieces with two contact points, which are driven by an 850 G linear actuator using an ESP300 motion controller manufactured by Newport. However, outer Al pieces with the other two contact points are fixed. Depending on which direction the inner part moves toward the cantilever, compressive or tensile stress can be introduced to the sensors.

At first, the influence of mechanical stress on the R(H) loops was studied on a round TMR sensor with a diameter of 30±0.2μm. The magnetic field was swept perpendicular to the induced anisotropy of the sense layer ($H⊥K_{u}$), as shown in Figure 2b, while the tunnel resistance of the wire-bonded sensor was measured under 10 mV voltage by a Keithley 2400 source meter. R(H) loops were measured under 0 to $0.76\times 10^{-3}$ strain values. The strain variation leads to different resistance changes $\Delta R_{\varepsilon }$ at different magnetic fields. This allows one to figure out at which bias fields the strain variation is accompanied by the maximum resistance change. Moreover, R(ε) loops were measured by continuously ramping up the strain to ∼$1\times 10^{-3}$ and recording the sensor resistance at the bias field with the maximum $\Delta R_{\varepsilon }$. From these loops, strain sensitivity of the TMR sensor was determined by measuring its gauge factors GF, defined by the relative change of the resistance as a function of the applied strain (GF=(ΔR(ε)/R)/ε).

## 3. Results and Discussion

### 3.1. Modeling of Field Loops

**Figure 3:**
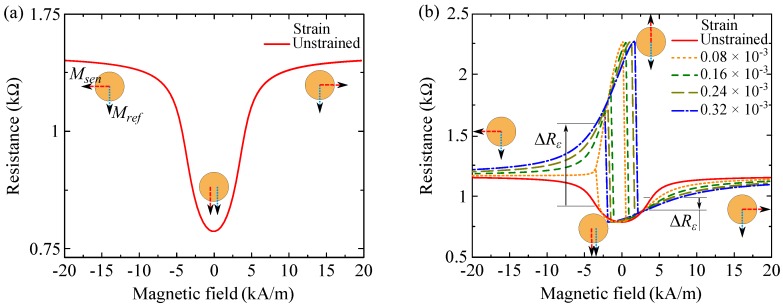
(**a**) Calculated R(H) loop in the unstrained state as the magnetic field is perpendicular to the induced anisotropy ($H⊥K_{u}$); (**b**) Changes in the R(H) loop imposed by different levels of tensile strain. Applying tensile strain leads to changes in the resistance ($\Delta R_{\varepsilon }$) at different magnetic fields.

Figure 3a shows a calculated field loop of a TMR junction in the unstrained state as the magnetic field is perpendicular to the induced anisotropy. During the loop, the magnetization of the sense layer rotates through the magnetization of the reference layer. In other words, the configuration between the two magnetizations varies from perpendicular at the highest negative field, to parallel at zero field, and then again to perpendicular at the highest positive field. Such rotation through the parallel state emanates from the Néel coupling, which acts as a unidirectional anisotropy $K_{nc}$ parallel to $M_{ref}$ [22].

Figure 3b demonstrates the successive changes of the R(H) loop, in Figure 3a, imposed by different levels of tensile strain. For $\varepsilon >0.08\times 10^{-3}$ the rotation tendency of $M_{sen}$ alters as the Néel coupling ($E_{nc}$ = 645 J/m${}^{3}$) is overcome due to further increase of the stress-induced anisotropy $K_{\sigma }$. For larger Néel couplings, the change in the rotation of $M_{sen}$ occurs at higher strain levels. The effective anisotropy influenced by both $K_{u}$ and $K_{\sigma }$ leads to switching fields into the field loop. The resistance changes abruptly at these switching fields. Within the magnetization reversal, the antiparallel configuration is now approached. The rotation mechanism of $M_{sen}$ in such field loops is thoroughly described with a macrospin model supported by micromagnetic simulation in our earlier study [3]. An increase of tensile strain results in more squareness of the field loop and larger switching fields. This is attributed to a higher stress-induced anisotropy $K_{\sigma }$ which magnetically stabilizes $M_{sen}$ more along the stress axis.

Under compressive stress, all physical quantities in Equation (2) will have the same magnitude and orientation as under tensile stress, except the axis of the stress-induced anisotropy which rotates by $\frac{\pi }{2}$ and aligns at $\theta_{\sigma }=\frac{\pi }{4}$. Therefore, the calculated field loops under compressive stress (σ<0) will be exactly the same but mirrored compared to the calculated field loops (Figure 3b) obtained under tensile stress.

### 3.2. Modeling of Strain Loops

**Figure 4:**
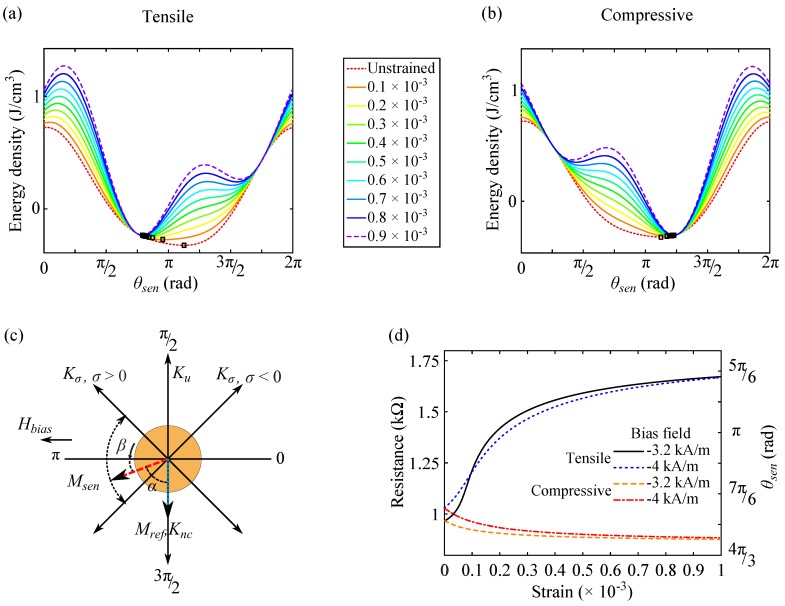
Orientation of $M_{sen}$, squares at minimum points in the energy density profiles of the sense layer at −3.2 kA/m (−40 Oe) under (**a**) tensile and (**b**) compressive stresses; (**c**) Configuration of physical quantities under tensile and compressive stresses; (**d**) Calculated strain loops at bias fields of −3.2 kA/m and −4 kA/m (−50 Oe). The right y-axis indicates the corresponding orientation of $M_{sen}$ extracted from the energy density profiles. Tensile (compressive) stress increases (reduces) the angle *α* resulting in a rise (drop) in the tunnel resistance.

Figure 4a,b demonstrates the evolution of energy density profiles of the sense layer under tensile and compressive mechanical stresses, respectively. Following the minor loop in the unstrained state in Figure 3a, prior saturation and then gradual field reduction to a bias field with a large $\Delta R_{\varepsilon }$ (Figure 3b) lead to a slight tilt *β* of $M_{sen}$ toward $M_{ref}$ caused by an interplay of the bias field and the Néel coupling ($\alpha =\frac{\pi }{2}-\beta$). This initial orientation of $M_{sen}$ can be determined by the minimum energy point in the unstrained state, as $\theta_{sen}=\pi +\beta$ in Figure 4c. With $H_{bias}=-3.2$ kA/m and $E_{nc}=645$ J/m${}^{3}$, *β* is about $\frac{\pi }{8}$ ($22.5^{\circ }$). Under tensile and compressive stresses $M_{sen}$ rotates toward the stress-induced anisotropy axis of $\theta_{\sigma }=\frac{3\pi }{4}$ and $\theta_{\sigma }=\frac{\pi }{4}$, respectively, as indicated in Figure 4c. The calculated strain loops are shown in Figure 4d.

They are defined as two regimes with different rates for the resistance change. Up to $0.2\times 10^{-3}$ strain, gauge factors of 2294 and −311 are calculated at $H_{bias}=-3.2$ kA/m under tensile and compressive stresses, respectively. Change of the bias field to −4 kA/m affects the rate of the resistance change in the strain loops leading to gauge factors of 1554 and −441. This points out the magnetic field dependence of the strain sensitivity.

Due to the symmetry of the physical quantities in Figure 4c, calculated strain loops at 3.2 kA/m and 4 kA/m bias fields will be the same as the strain loops at −3.2 kA/m and −4 kA/m bias fields (Figure 4d). However, tensile strain loops will be switched to compressive strain loops, and vice versa.

### 3.3. Mechanical Stress Influence on R(H) Loops

**Figure 5:**
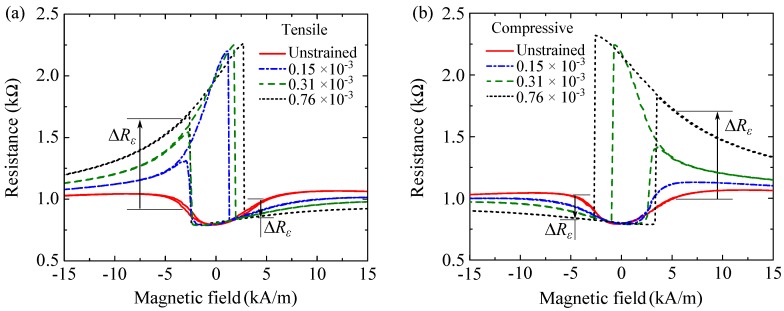
Applying (**a**) tensile ($K_{\sigma }$, $\theta_{\sigma }=\frac{3\pi }{4}$) and (**b**) compressive stresses ($K_{\sigma }$, $\theta_{\sigma }=\frac{\pi }{4}$) imposes deformation into the R(H) loop of the junction in the unstrained state. The red plots represent the R(H) loop in the unstrained state.

As shown in Figure 5a,b, the R(H) loop of the junction in the unstrained state changes consecutively under different strain levels. Increasing the strain further enlarges the switching fields as in the calculated R(H) loops in Figure 3b. It also reduces the asymmetry of the field loop. In contrast to the modeling results, higher strain gradually increases the highest resistance measured in the field loop, which becomes nearly $R_{ap}$ ($\theta_{sen}=\frac{\pi }{2}$). Also, $R_{p}$ ($\theta_{sen}=\frac{3\pi }{2}$) is gradually reached within the field loop. This difference can be explained by caned magnetization and domain formation in the sense layer which are not considered in the modeling under the assumption of single-domain behavior. In comparison to tensile stress shown in Figure 5a, under compressive stress, the corresponding stress-induced anisotropy is perpendicular to the axis of the applied stress. As a result, similar but mirrored R(H) loops are observed (Figure 5b).

With the junction strained up to $0.76\times 10^{-3}$, the large resistance increase $\Delta R_{\varepsilon }$ in case of tensile stress occurs at negative fields, H> −3 kA/m, reaching the maximum near the switching fields (Figure 5a). However, as for positive fields, H> +3 kA/m, the resistance slightly decreases. The strain effect is mirrored for compressive stress (Figure 5b) since the uniaxial stress-induced anisotropy resides along an axis perpendicular to the one induced by tensile stress. Consequently, under compressive stress the large resistance increase and the small resistance decrease take place at positive H> +3 kA/m and negative fields H> −3 kA/m, respectively.

### 3.4. Strain Sensitivity

**Figure 6:**
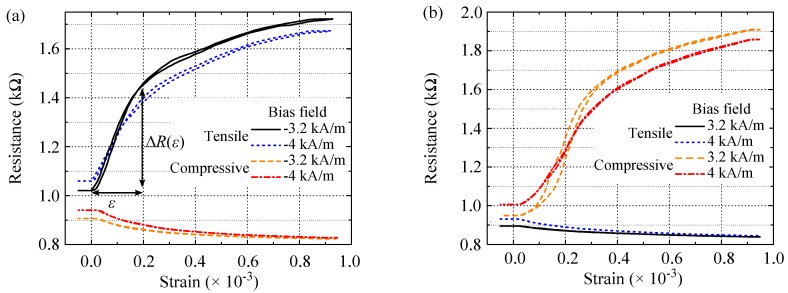
Strain loops of the junction measured in the presence of (**a**) negative and (**b**) positive bias fields. ΔR(ε) stands for the resistance change in the strain loops, associated with the applied strain *ε*. Panel (**a**) is partially reproduced from [22] with permission from Elsevier.

Figure 6a,b demonstrates strain loops of the junction at ±3.2 kA/m (±40 Oe) and ±4 kA/m (±50 Oe) bias fields. The bias fields are chosen to be away from the switching fields at which small strain variations lead to abrupt and hysteretic magnetization and resistance changes.

As expected from Figure 5a,b and the modeling results, applying tensile stress leads to a large resistance increase at negative bias fields whereas under compressive stress the resistance slightly decreases. The impact on the resistance is reversed between tensile and compressive stresses. Gauge factors measured at the first part of the loops up to $\varepsilon =0.2\times 10^{-3}$ are listed in Table 2. Different gauge factors obtained at different bias fields indicate a strong field dependence of the strain sensitivity. The field dependence is also present in the calculated strain loops shown in Figure 4d. It is attributed to the initial magnetization orientation of the sense layer $\theta_{sen}$, which can be tuned by the bias field. As a result, on one hand, different bias fields affect the configuration between $M_{sen}$ and $k_{\sigma }$ leading to a different influence on the angle *α* and the resistance by mechanical stress. On the other hand, the bias field sets the initial angle *α* between the magnetization of the two electrodes. This contributes to the magnitude of the resistance change since the change rate of the resistance $\frac{dR(\alpha )}{d\alpha }$, the first derivation of Equation (1), is angular dependent. For this sensor, the maximum gauge factor of GF=2150±30 under tensile stress was obtained at the −3.2 kA/m bias field. In comparison, compressive stress leads to a much smaller gauge factor of −260. The calculated strain loops in Figure 4d have nearly the same trend and gauge factors. At the positive bias field $H_{bias}=$ +4 kA/m, the compressive strain loop, exhibits the maximum gauge factor of GF=1750±35 whereas a low gauge factor of −250 was measured under tensile stress. The hysteresis in the compressive strain loop at $H_{bias}=+3.2$ kA/m is attributed to the bias field being between the switching fields of the R(H) loops in Figure 5b. Therefore, the gauge factor of the compressive loop at this bias field is not given in Table 2.

**Figure 7:**
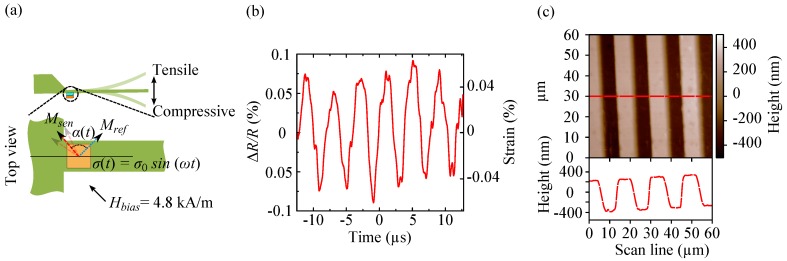
(**a**) Oscillation of an AFM cantilever at its resonance frequency; (**b**) The increase and the decrease in the tunnel resistance are correlated to the stress alternation. The oscillating signal or ΔR/R is detected using a manually balanced Wheatstone bridge configuration, in which the TMR sensor is incorporated; (**c**) Dynamic imaging of a PMMA grating in amplitude modulation mode recorded by a TMR sensor. Panel (**c**) is reproduced from [6] with permission of AIP Publishing.

The inverse impact on the resistance by tensile and compressive stresses gives the possibility to distinguish between both stresses, even though the gauge factors are not equal at these bias fields. As shown in Figure 7, this achievement extends the application of such sensors to dynamic devices e.g., dynamic mode AFM [15]. As a TMR-based AFM cantilever is oscillated at its resonance frequency (Figure 7a), upward and downward bending causes stress alternation σ(t) between tensile and compressive stresses. Therefore, $M_{sen}$ oscillates at its initial orientation set by $H_{bias}$ = 4.8 kA/m. Consequently, the angle *α* varies accordingly (α(t)) resulting in oscillation of the tunnel resistance as shown in Figure 7b. Reading out the cantilever deflection by TMR sensors in the dynamic mode approves their reliability as an alternative for the optical read-out in AFM measurements. Figure 7c shows a topography image of a PMMA grating obtained by dynamic imaging using a TMR sensor read-out [6]. Having studied TMR sensors in terms of minimum detectable deflection as height and phase contrasts, atomic-step edges of 2.54 Å on Au (111) terraces and self-assembled monolayers of peruorodecyltrichlorosilane have been successfully imaged by amplitude and frequency modulated AFM [15].

## 4. Conclusions

We demonstrated numerical simulations based on a modified SW model to calculate field and strain loops with the proposed configuration ($∠\left(H,K_{u}\right)=\frac{\pi }{2}$ and $∠\left(\sigma ,K_{u}\right)=\frac{\pi }{4}$). Using the energy minimization, the rotation of $M_{sen}$ in the tensile and compressive strain loops at $H_{bias}=-3.2$ kA/m are successfully described. As a result, the inverse impact of tensile and compressive stresses on the tunnel resistance is approved by the calculated strain loops.

Investigation by the modeling was also carried out experimentally on a round junction with a diameter of 30±0.2 μm. A certain agreement prevails in the experimental and modeling results. The measured field and strain loops exhibit nearly the same trends as the calculated ones. Also, the gauge factors measured experimentally from the strain loops are in the range of the calculated gauge factors. Measured tensile and compressive strain loops, at ±3.2 kA/m and ±4 kA/m bias fields, reveal the strain sensitivity dependence on the bias field and the inverse impact on the tunnel resistance by both stresses. At −3.2 kA/m, the junction exhibits GF=2150±30 for tensile stress whereas for compressive stress the gauge factor of −260 was measured. Conversely, at +4 kA/m, a positive bias field, the junction shows GF=1750±35 and GF=-250 for compressive and tensile stresses, respectively. Despite the unequal gauge factors at a bias field, the inverse impact by tensile and compressive stresses on the tunnel resistance allows detection of both stresses by a single sensor. The potential of such miniaturized highly sensitive sensors is demonstrated by their AFM applications. However, the necessity of an external bias magnetic field during operation is recognized as their technical drawback in terms of a simple and compact strain sensing setup. In order to avoid the use of the external magnetic field by magnetic coils, ongoing research is in progress with a view of integrating permanent micromagnets or internal magnetic biasing. 

## Figures and Tables

**Table 1:** Material parameters used for the modified SW model.

Physical Quantity	Value
Saturation magnetization ($M_{s}$)	1030 kA/m
Induced anisotropy ($K_{u}$)	2050 J/m${}^{2}$
Young’s modulus (*Y*)	169 GPa
Isotropic magnetostriction ($\lambda_{s}$)	$30\times 10^{-6}$ [23]

**Table 2:** Gauge factors GF measured from the strain loops in Figure 6. The positive and negative signs of the gauge factors stand for the resistance increase and decrease in the strain loops.

*H*${}_{\mathrm{bias}}$(kA/m)		−3.2	−4	3.2	4
***GF***	**Tensile**	2150±30	1550±25	−150	−250
	**Compressive**	−260	−335	-	1750±35

## Abbreviations

The following abbreviations are used in this manuscript:

TMR	Tunnel magnetoresistance
MTJ	Magnetic tunnel junction
SW	Stoner–Wohlfarth
MR	Magnetoresistance
AFM	Atomic force microscopy

## References

[1] Tadigadapa S., Mateti K. (2009). Piezoelectric MEMS sensors: State-of-the-art and perspectives. Meas. Sci. Technol..

[2] Barlian A.A., Park W.-T., Mallon J.R., Rastegar A.J., Pruitt B.L. (2009). Review: Semiconductor piezoresistance for microsystems. Proc. IEEE.

[3] Tavassolizadeh A., Hayes P., Rott K., Reiss G., Quandt E., Meyners D. (2015). Highly strain-sensitive magnetostrictive tunnel magnetoresistance junctions. J. Magn. Magn. Mater..

[4] Sahoo D.R., Sebastian A., Häberle W., Pozidis H., Eleftheriou E. (2011). Scanning probe microscopy based on magnetoresistive sensing. Nanotechnology.

[5] Meyners D., von Hofe T., Vieth M., Rührig M., Schmitt S., Quandt E. (2009). Pressure sensor based on magnetic tunnel junctions. J. Appl. Phys..

[6] Tavassolizadeh A., Meier T., Rott K., Reiss G., Quandt E., Hölscher H., Meyners D. (2013). Self-sensing atomic force microscopy cantilevers based on tunnel magnetoresistance sensors. Appl. Phys. Lett..

[7] O’Handley R.C., Chlidress J.R. (1995). New spin-valve magnetic field sensors combined with strain sensing and strain compensation. IEEE Trans. Magn..

[8] Mamin H.J., Gurney B.A., Wilhoit D.R., Speriosu V.S. (1998). High sensitivity spin-valve strain sensor. Appl. Phys. Lett..

[9] Baril L., Gurney B., Wilhoit D., Speriosu V.S. (1999). Magnetostriction in spin valves. J. Appl. Phys..

[10] Duenas T., Sehrbrock A., Löhndorf M., Ludwig A., Wecker J., Grünberg P., Quandt E. (2002). Micro-sensor coupling magnetostriction and magnetoresistive phenomena. J. Magn. Magn. Mater..

[11] Wang D., Nordman C., Qian Z., Daughton J.M., Myers J. (2005). Magnetostriction effect of amorphous CoFeB thin films and application in spin-dependent tunnel junctions. J. Appl. Phys..

[12] Löhndorf M., Duenas T., Tewes M., Quandt E. (2002). Highly sensitive strain sensors based on magnetic tunneling junctions. Appl. Phys. Lett..

[13] Löhndorf M., Duenas T., Ludwig A., Rührig M., Wecker J., Bürgler D., Grünberg P., Quandt E. (2002). Strain sensors based on magnetostrictive GMR/TMR structures. IEEE Trans. Magn..

[14] Löhndorf M., Dokupil S., Bootsmann M.-T., Malavé A., Rührig M., Bär L., Quandt E. (2007). Characterization of magnetostrictive TMR pressure sensors by MOKE. J. Magn. Magn. Mater..

[15] Meier T., Förste A., Tavassolizadeh A., Rott K., Meyners D., Gröger R., Reiss G., Quandt E., Schimmel T., Hölscher H. (2015). A scanning probe microscope for magnetoresistive cantilevers utilizing a nested scanner design for large-area scans. Beilstein J. Nanotechnol..

[16] Bootsmann M.-T., Dokupil S., Quandt E., Ivanov T., Abedinov N.C., Löhndorf M. (2005). Switching of magnetostrictive micro-dot arrays by mechanical strain. IEEE Trans. Magn..

[17] Jaffrès H., Lacour D., Nguyen Van Dau F., Briatico J., Petroff F., Vaurès A. (2001). Angular dependence of the tunnel magnetoresistance in transition-metal-based junctions. Phys. Rev. B.

[18] Xu X., Li M., Hu J., Dai J., Xia W. (2010). Strain-induced magnetoresistance for novel strain sensors. J. Appl. Phys..

[19] Hauser H., Rührig M., Wecker J. (2004). Hysteresis modeling of tunneling magnetoresistance strain sensor elements. J. Appl. Phys..

[20] Meyners D., Puchalla J., Dokupil S., Löhndorf M., Quandt E. (2007). Magnetoelectrical sensors for mechanical measurements. ESC Trans..

[21] Lebedev G.A., Viala B., Lafont T., Zakharov D.I., Cugat O., Delamare J. (2012). Converse magnetoelectric effect dependence with CoFeB composition in ferromagnetic/piezoelectric composites. J. Appl. Phys..

[22] Tavassolizadeh A. (2016). Miniaturized Tunnel Magnetoresistance Sensors for Novel Applications of Atomic Force Microscopy. Ph.D. Dissertation.

[23] Platt C.L., Minor M.K., Klemmer T.J. (2001). Magnetic and structural properties of FeCoB thin films. IEEE Trans. Magn..

